# Long non-coding RNA PTENP1 functions as a ceRNA to modulate PTEN level by decoying miR-106b and miR-93 in gastric cancer

**DOI:** 10.18632/oncotarget.15317

**Published:** 2017-02-13

**Authors:** Rupeng Zhang, Yuenan Guo, Zhenchi Ma, Gang Ma, Qiang Xue, Fangxuan Li, Liren Liu

**Affiliations:** ^1^ Department of Gastric Cancer Surgery, Tianjin Medical University Cancer Institute and Hospital, Tianjin 300060, China; ^2^ Department of Gastrointestinal Cancer Biology, Tianjin Medical University Cancer Institute and Hospital, Tianjin 300060, China; ^3^ Department of Cancer Prevention Center, Tianjin Medical University Cancer Institute and Hospital, Tianjin 300060, China; ^4^ National Clinical Research Center of Cancer, Tianjin 300060, China; ^5^ Tianjin's Clinical Research Center for Cancer, Tianjin 300060, China

**Keywords:** PTENP1, miR-106b, miR-93, long non-coding RNA, gastric cancer

## Abstract

Recent studies have shown that competing endogenous RNAs (ceRNAs) play an important role in the regulation of gene expression, and participate in a wide range of biological processes, including carcinogenesis. Long non-coding RNA PTENP1, the pseudogene of PTEN tumor suppressor, has been reported to exert its tumor suppressive function via modulation of PTEN expression in many malignancies. However, whether a PTENP1∼miRNA∼PTEN ceRNA network exists and how it functions in gastric cancer (GC) remains elusive. In order to identify and characterize the PTENP1∼miRNA∼PTEN ceRNA network in GC, we first determined PTENP1 levels in clinical GC samples and found that PTENP1 and PTEN were concurrently downregulated in these samples. We further demonstrated that PTENP1 could act as a ceRNA to sponge miR-106b and miR-93 from targeting PTEN for downregulation using a novel ceRNA *in vitro* gradient assay. Thus, we revealed a tumor suppressive role of PTENP1 as ceRNA in GC and pinpointed the specific miRNAs decoyed by PTENP1, highlighting the emerging roles of ceRNAs in the biological regulation of GC cells and their possible clinical significance.

## INTRODUCTION

Gastric cancer (GC) remains a major global health problem, ranking the fifth most common malignancy and the third leading cause of cancer mortality worldwide [1]. Early diagnosis is critical to the prognosis of GC patients. However, approximately two thirds of patients are diagnosed at advanced stages, resulting in disappointing outcomes primarily due to a high recurrence rate [1]. Despite significant progress in the diagnosis of GC as well as surgical combined adjuvant approaches, the outcomes of GC patients have only modestly improved [2]. Therefore, a better understanding of the underlying molecular and biological mechanisms in GC carcinogenesis and progression may uncover novel therapeutics to treat this difficult disease.

It is known that discrete genetic alterations alone cannot fully expsdlain the complexity of GC pathogenesis, whereas dysregulated epigenetic mechanisms, such as altered non-coding RNA (ncRNA), have been recently implicated in the carcinogenesis and progression of GC. In general, ncRNAs are classified as long ncRNAs (lncRNAs, > 200 nt) and small ncRNAs (≤ 200 nt). LncRNAs consist of antisense, intronic, intergenic transcripts, and pseudogenes, accounting for a significant proportion of the “transcriptome”. Recently, a new role for pseudogenes was discovered which identified a competitive endogenous RNA (ceRNA) that functions to sequester the miRNAs from binding to their ancestral genes [3, 4]. PTENP1, the pseudogene of PTEN tumor suppressor, contains a highly homologous region upstream of the 3′UTR of PTEN. These highly conserved seed sequences that match PTEN-targeting miRNAs, downregulate PTEN by serving as a ceRNA to decoy the miRNAs. Recent reports have shown that PTENP1 is lost or downregulated concomitantly with PTEN in a number of malignancies [3, 5–9]. In line with this observation, ectopic expression of PTENP1 resulted in the upregulation of PTEN, accompanied by the blockage of PI3K/AKT pathway and growth inhibition in prostate (DU145) and renal (ACHN and SN12PM6) cancer cells [3, 6]. Specifically, PTENP1 can modulate PTEN levels by sponging miR21 in ACHN and SN12PM6 renal cancer cells, while exerting its decoy effect by trapping miR17, miR19b and miR20a, which would otherwise target PTEN and several autophagy gene transcripts in Mahlavu HCC cells. Thus, PTENP1 exerts its tumor suppressive function as a ceRNA through distinct molecular mechanisms in different cancer types.

In this study, we determined the tumor suppressive role of PTENP1 as a ceRNA in GC and identified the specific miRNAs decoyed by PTENP1, highlighting the emerging roles of ceRNAs in the biological regulation of GC cells and their possible clinical significance.

## RESULTS

### PTENP1 and PTEN are concomitantly downregulated in human GC tissues

**Figure 1:**
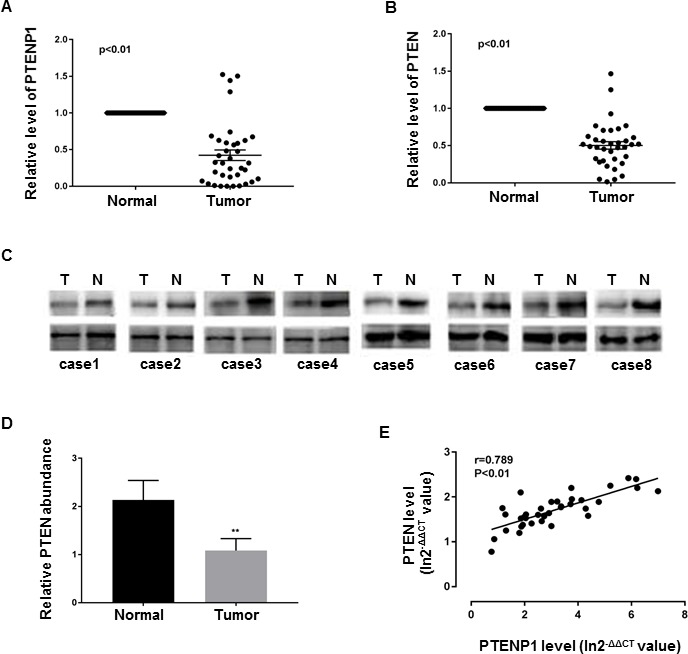
PTEN and PTENP1 were concurrently downregulated in GC (**A**–**B**) PTENP1 and PTEN mRNA levels were evaluated by qRT-PCR in paired GC samples. Data are presented as 2^−ΔΔCT^. (**C**–**D**) PTEN protein was determined by western blot. Each assay was done in triplicate. *P* values were obtained by paired *t-test* (**P < 0.01). (**E**) The relationship between PTEN and PTENP1 mRNA were analyzed by linear regression analysis. Data are presented as In 2^−ΔΔCT^.

Studies have found PTENP1 to be lost or downregulated in various cancers [3, 5, 6, 8, 10, 11]. In ordert to evaluate PTENP1 status in GC, we determined the levels PTENP1 transcripts by qRT-PCR in 36 GC biopsies and their paired adjacent normal tissues from the same patients. We found that 88.9% (32 out of 36) had approximate 2-fold lower mRNA levels of PTENP1 in GC tissues relative to the paired normal tissues (*p* < 0.01) (Figure 1A). We next determined the expression of PTEN, the parental gene of PTENP1, in the same paired biopsies. As expected, we found that 94.4% (34 out of 36) GC samples had about 2-fold decreased PTEN mRNA levels, which was further confirmed by immunoblotting (Figure 1B–1D). Performing linear regression analysis demonstrated that there was a positive correlation between PTENP1 and PTEN downregulation in GC tissues (rs = 0.7889, *p* < 0.05) (Figure 1E).

### PTENP1 and PTEN expression levels were associated with the pathological features of GC patients

We next examined the significance of PTENP1 and PTEN expression correlated to the pathological features of GC patients (Table 1). There were no remarkable differences in PTENP1 or PTEN expression between different patient demographics, while there were significant differences observed in the distribution of the tumor size (PTENP1: *p* = 0.031; PTEN: *p* = 0.027), clinic stage (PTENP1: *p* = 0.025; PTEN: *p* = 0.002) and invasion depth (PTENP1: *p* = 0.001; PTEN: *p* = 0.008). Although no statistical difference of distant metastasis was found, the PTENP1 or PTEN downregulated patients showed significant and more frequent lymph node metastasis (PTENP1: *p* = 0.032; PTENP1: *p* = 0.018), indicating PTENP1 or PTEN could be a potential valuable biomarker for predicting lymph node metastasis in GC patients (Supplementary Figure 1).

**Table 1:** PTENP1 and PTEN expression levels were associated with the pathological features of GC patients

Factors	Case	PTENP1 (mean)	*P* value	PTEN (mean)	*P* value
Gender			0.489		0.401
Male	23	2.962 ± 1.49		1.662 ± 0.37	
Female	13	3.351 ± 1.79		1.772 ± 0.37	
Age(years)			0.360		0.079
< 60	22	3.299 ± 1.64		1.788 ± 0.37	
≥ 60	14	2.793 ± 1.51		1.566 ± 0.34	
Tumor size (cm)			0.031		0.027
≥ 5	26	2.262 ± 0.92		1.619 ± 0.27	
< 5	10	5.108 ± 1.10		1.918 ± 0.50	
Histological grade			0.549		0.452
Well-intermediately differentiation	15	2.911 ± 1.55		1.646 ± 0.33	
Poor differentiation	21	3.239 ± 1.64		1.741 ± 0.40	
Invasion depth			0.001		0.008
T1–T2	6	5.032 ± 1.92		2.057 ± 0.32	
T3–T4	30	2.716 ± 1.22		1.631 ± 0.34	
Lymph node metastasis			0.032		0.018
N0	7	4.784 ± 1.98		1.993 ± 0.41	
N1–N3	29	2.696 ± 1.20		1.631 ± 0.33	
Distant metastasis			0.478		0.656
M0	4	2.560 ± 1.17		1.623 ± 0.22	
M1	32	3.170 ± 1.64		1.712 ± 0.39	
TNM stage			0.025		0.002
I, II	8	4.695 ± 2.04		2.046 ± 0.35	
III, IV	28	2.647 ± 1.11		1.603 ± 0.31	

### PTENP1 modulated PTEN expression in GC cells

**Figure 2:**
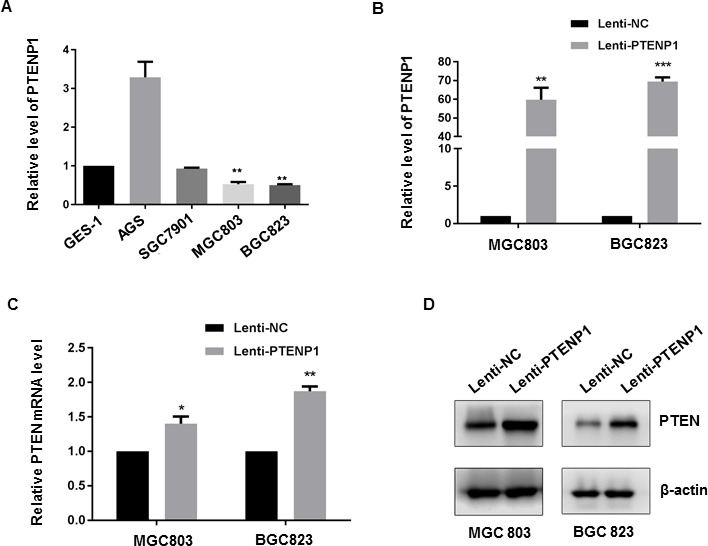
PTENP1 upregulated PTEN expression in GC cells (**A**) PTENP1 expression level in GC cells were analyzed by qRT-PCR compared to GES-1. (**B**) Lentiviral mediated expression of PTENP1 was validated by qRT-PCR in GC cells. (**C**–**D**) PTEN mRNA (C) and protein level (D) were determined after overexpression of PTENP1 in MGC803 and BGC823 cells by qRT-PCR and western blot, respectively. *P* values were obtained by paired *t-test* (*P < 0.05; **P < 0.01; ***P < 0.001).

Having validated the downregulation of PTENP1 in clinical GC samples, we next examined PTENP1 expression in different human GC cell lines (AGS, SGC7901, MGC803 and BGC823) as well as human gastric epithelial mucosa cells (GES-1). As shown in Figure 2A, the abundance of PTENP1 was significantly lower in MGC803 and BGC823 cells than in GES-1 cells, whereas relative high PTENP1 expression was observed in AGS cells. In order to determine the role of PTENP1 in modulating PTEN level in GC cells, we ectopically over-expressed Lenti-PTENP1 in the PTENP1 low-expressing MGC803 and BGC823 cells. Stable over-expression of PTENP1 was verified by qRT-PCR (Figure 2B) and resulted in the upregulation of PTEN expression at both the mRNA and the protein level in MGC803 and BGC823 cells (Figure 2C–2D). Thus, the expression level of PTEN in GC cells was modulated by PTENP1.

### PTENP1 overexpression inhibited cell growth and induced apoptosis in GC cells

**Figure 3:**
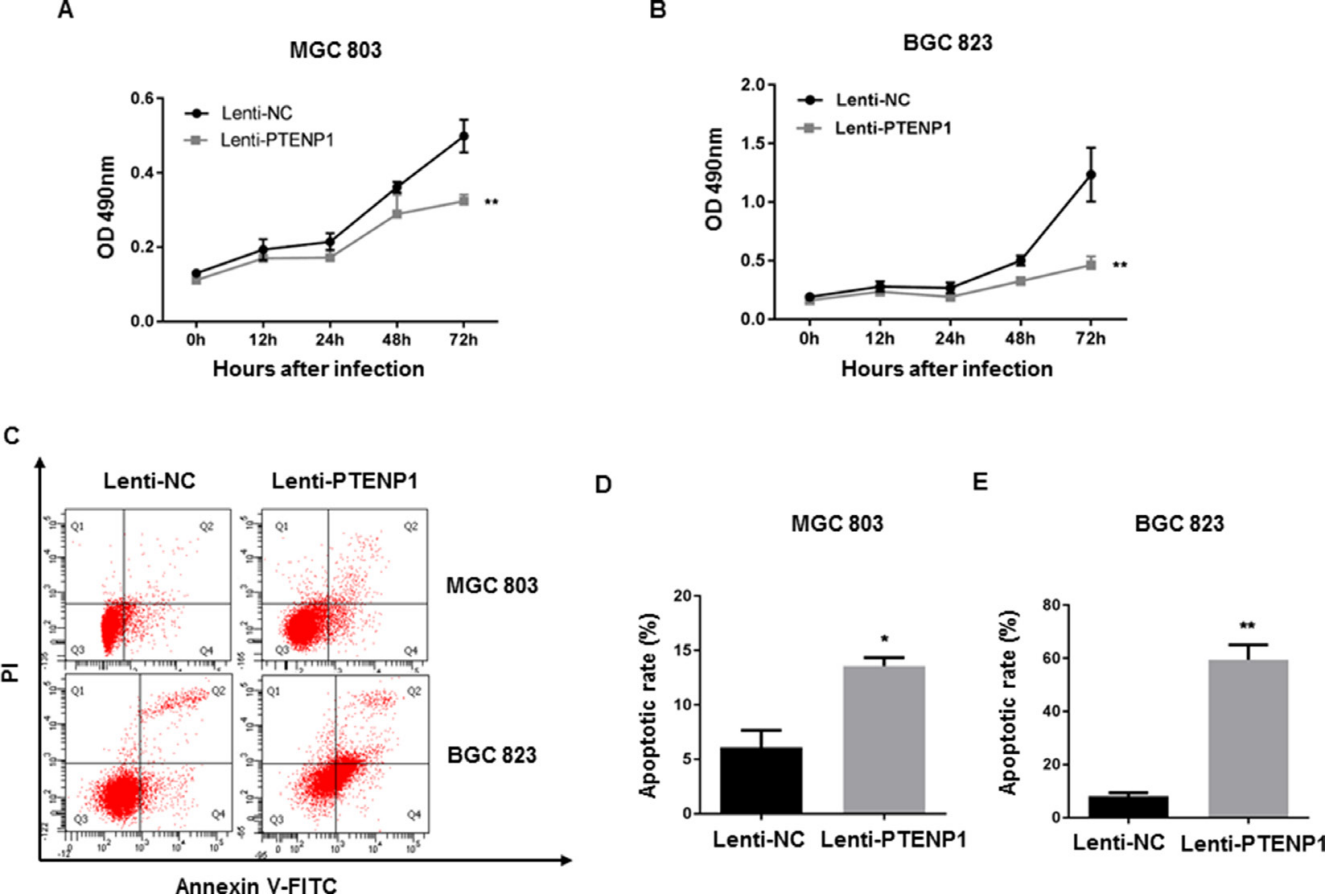
PTENP1 inhibited cell proliferation and induced apoptosis (**A**–**B**) Cell proliferation in response to overexpression of PTENPI was evaluated at different time points by MTT assay in GC cells. (**C**) The transfected cells were harvested and stained with Annexin-V-FITC/PI to examine apoptosis by flow cytometry. (**D**–**E**) The percentages of apoptotic cells were defined as early apoptosis (Q4) + late apoptosis (Q2).

In order to explore the functional significance of PTENP1 upregulation on cell regulation and growth we performed an MTT assay and found that PTENP1 over-expressing MGC803 and BGC823 cells grew relatively slower than control (empty) lentivirus vectors, indicating that PTENP1 overexpression can inhibit cell growth (Figure 3A–3B). Since apoptosis is a critical factor leading to cell growth inhibition, we examined apoptosis using annexin V-FITC/PI labeling via flow cytometry. As shown in Figure 3C, overexpression of PTENP1 increased both early and late apoptotic events in GC cells. The total PTENP1-induced apoptotic population increased by nearly 3- and 7-fold in MGC803 and BGC823 cells compared to the control groups, respectively (Figure 3D–3E).

### Overexpression of PTENP1 suppresses migration and invasion of GC cells

**Figure 4:**
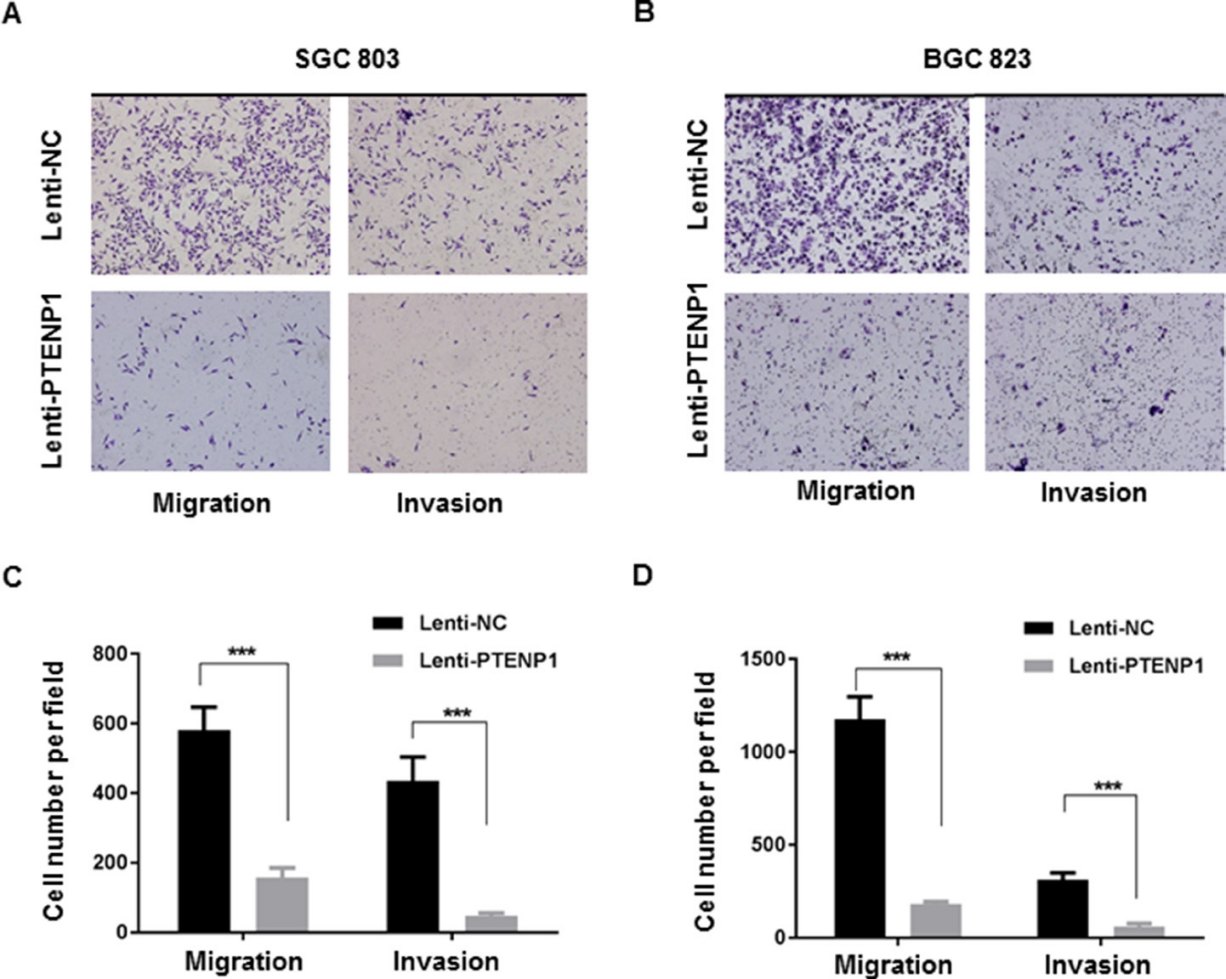
Overexpression of PTENP1 inhibited migratory and invasive ability of MGC803 and BGC823 cells (**A**–**B**) Representative photographs of cells passing through the membranes with Matrigel-coated or uncoated were taken at 200× magnification. (**C**–**D**) Migrated and invaded cells were quantified by counting in 10 random fields at 200× magnification. *P* values were obtained by paired *t-test* (***P < 0.001).

Cellular migration and invasion play important roles in cancer progression. In order to investigate the impact of PTENP1 overexpression on GC cell migration and invasion, we examined the ability of PTENP1 overexpression in GC cells to affect migration through uncoated and matrigel-coated transwell membranes. In agreement with our histopathological data that showed that downregulated PTENP1 was associated with bigger tumor size and more frequent lymph node metastasis, our transwell migration assay showed that high PTENP1 levels impeded cell migration and invasion, further demonstrating a tumor suppressive role of PTENP1 in GC cells (Figure 4A–4D).

### PTENP1 decoyed miR-106b and miR-93 from binding to PTEN mRNA in GC cells

**Figure 5:**
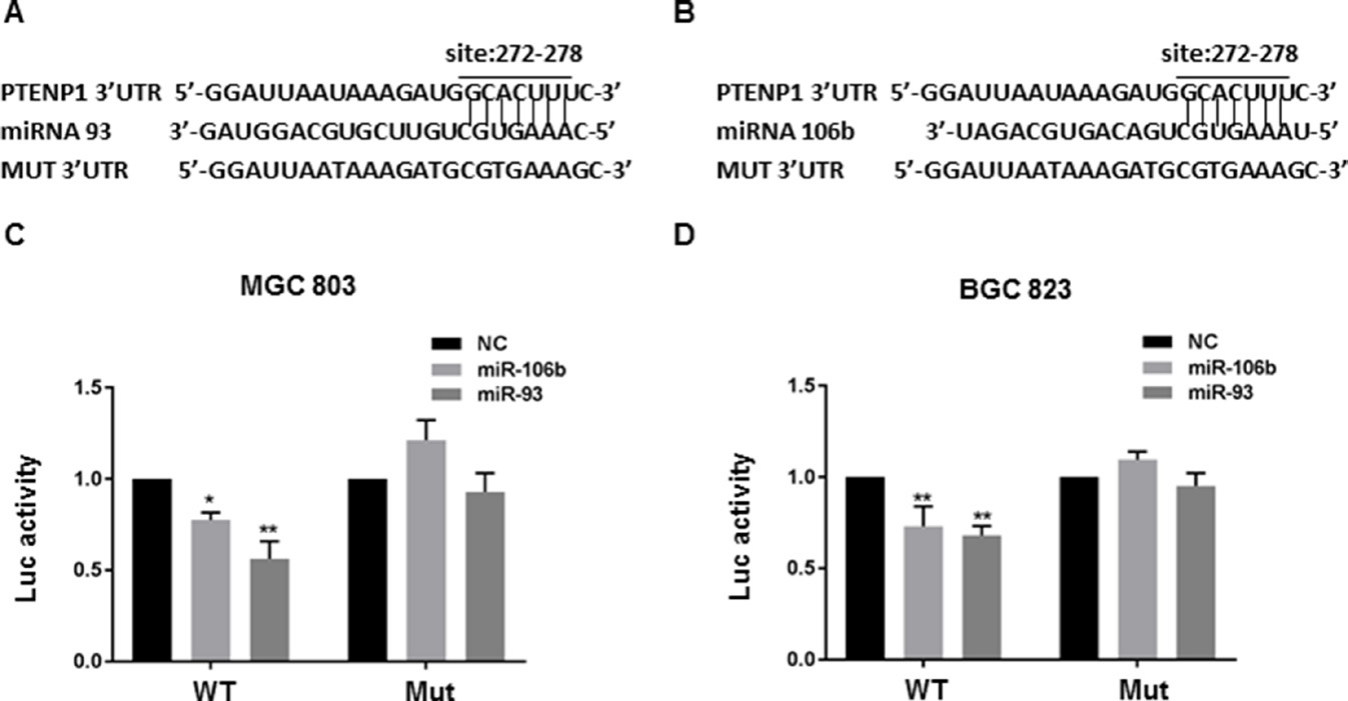

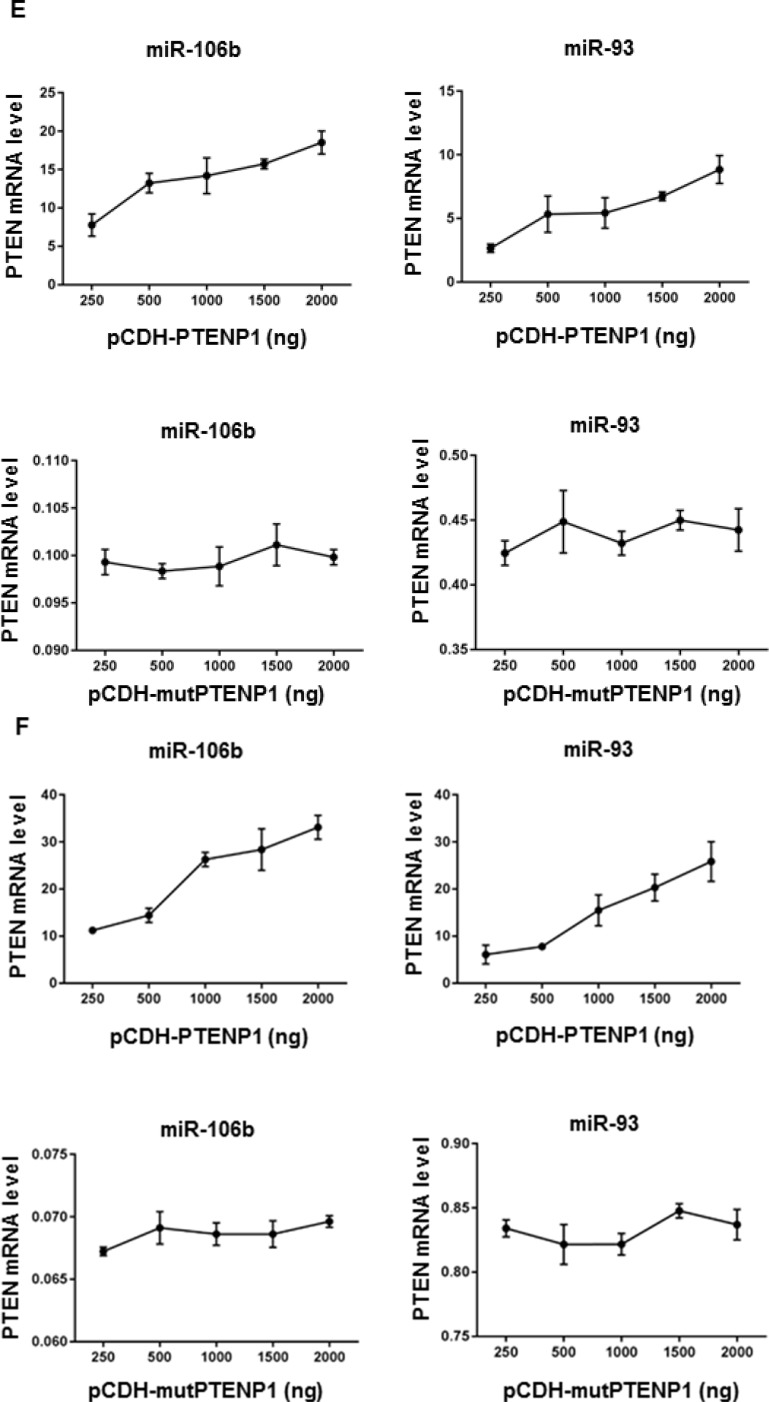
PTENP1 modulated PTEN level by competitively binding to miR-106b/miR-93 (**A**–**B**) The sequences of PTENP1 3′UTR, miR-106b/miR-93, and mutant PTENP1 3′UTR were shown. (**C**–**D**) pIS0-PTENP1 or pIS0-mutPTENP1 were co-transfected into MGC803 and BGC823 cells with miR-106b mimics, miR-93 mimics or negative control (NC). Luciferase signals were then detected after 24 hours. (**E**–**F**) Either miR-10b6 or miR-93 was co-transfected with consecutive increased doses of pCDH-PTENP1 (250 ng, 500 ng, 1000 ng, 1500 ng and 2000 ng) and pCDH-mutPTENP1 (250 ng, 500 ng, 1000 ng, 1500 ng and 2000 ng) into MGC803 (E) and BGC823 (F) cells, respectively. Then PTEN mRNA levels corresponding to different doses of pCDH-PTENP1 and pCDH-mutPTENP1were evaluated by qRT-PCR. Data are presented as 2^−ΔΔCT^.

The 3′UTR of the PTENP1 transcript contains numerous seed sequences that match for PTEN-targeting miRNAs. In order to identify the novel miRNA(s) that could bind to PTENP1 and PTEN transcripts simultaneously in GC cells, TargetScan, miRanda and Microcosm Targets bioinformatics software were employed (Supplementary Table 1). A seven base-pair sequence at position 272–278 of PTEN 3′UTR was identified to be the seed sequence for miR-106b/miR-93, which was further validated to be capable of targeting PTENP1 transcript by using Clustal Omega (Figure 5A–5B, Supplementary Figure 2). Using a luciferase reporter assay we revealed that ectopic expression of miR-106b or miR-93 mimics significantly decreases the luciferase activity of the reporter containing the wild-type 3′UTR of PTENP1, while had little or even opposing effects on the mutant bearing disrupted seed sequence for miR-106b/miR-93 (Figure 5C–5D). Thus, we conclude that PTENP1 is directly regulated by miR-106b and miR-93 in GC cells.

The PTEN ceRNA network (lncRNA∼miRNA∼mRNA) is complicated in that 3′UTR of PTEN contains various microRNA response elements (MREs) for multiple distinct miRNAs, and reciprocally, those miRNAs often target multiple distinct transcripts that may impact PTEN expression [12, 13]. In order to demonstrate that PTENP1 modulates PTEN expression via sponging miR-106b and miR-93 (miR-106b/miR-93∼PTEN ceRNA network), we carried out a novel ceRNA concentration gradient assay, in which a series of increasing amounts of the plasmid pCDH-PTENP1 or pCDH-mutPTENP1 containing disrupted seed sequence for miR-106b/miR-93 was co-transfected into GC cells with a constant amount of miR-106b or miR-93 mimics. After the cells were harvested at 48 hours post transfection, the PTEN levels were determined by qRT-PCR. As shown in Figure 5E–5F, the levels of PTEN transcripts increased as ectopic PTENP1 increased, exhibiting a linear dose-dependent pattern. Similar results were obtained by co-transfecting either miR-106b or miR-93 mimics. However, this PTENP1 dose-dependent PTEN expression was abrogated by disrupting the seed sequence for miR-106b/miR-93 within 3′UTR of PTENP1. Together these results demonstrated that PTENP1 modulated the expression of PTEN by competitively binding to miR-106b and miR-93 in GC cells.

### Correlation analysis of miR-106b/miR-93 and PTENP1/PTEN expression in GC tissues

**Figure 6:**
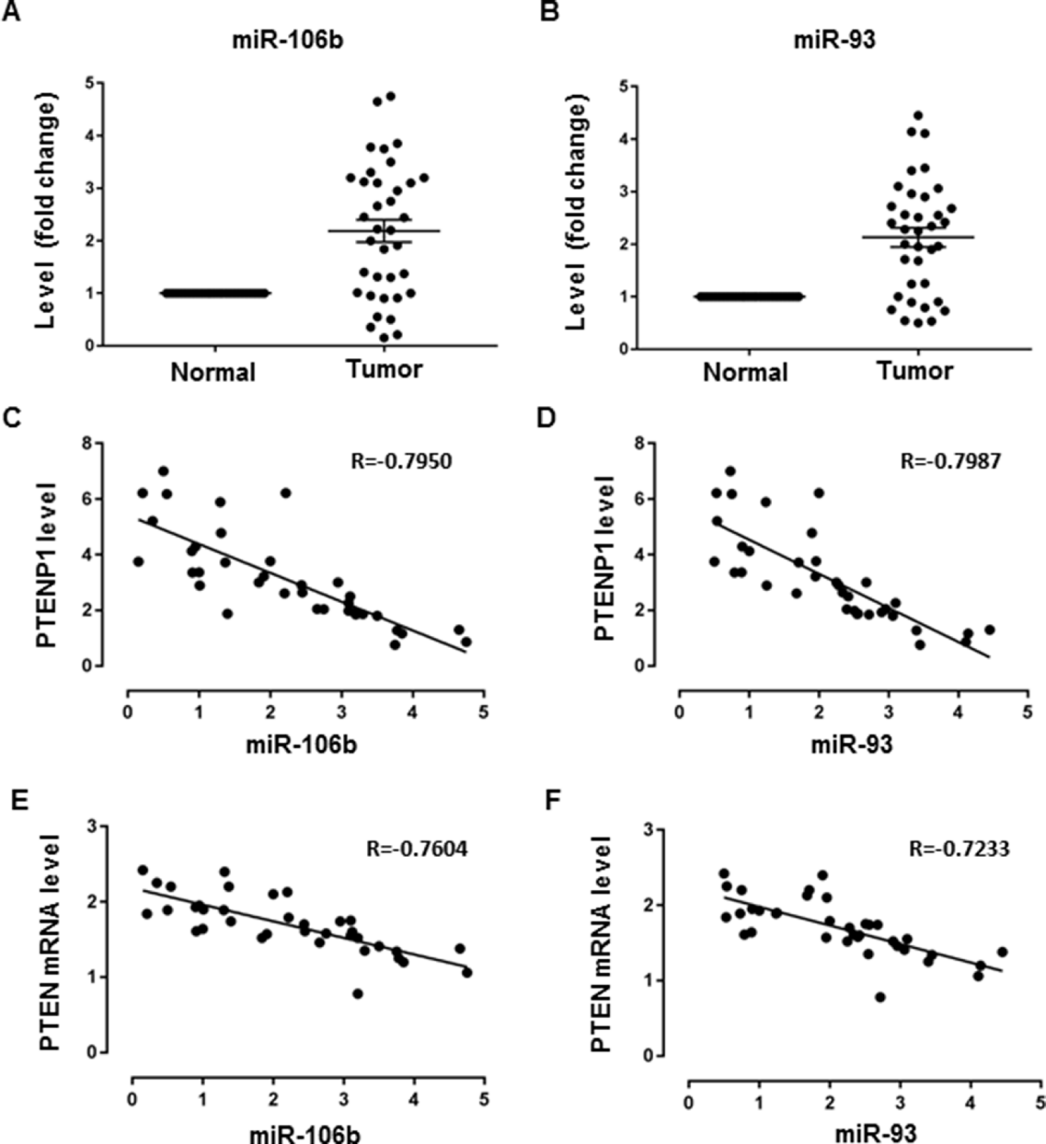
Correlation analysis of miR-106b/miR-93 and PTENP1/PTEN expression in GC (**A**–**B**) The levels of miR-106b and miR-93 in 36 GC samples relative to the paired normal controls were evaluated by qRT-PCR in paired GC samples. Data are presented as 2^−ΔΔCT^. (**C**–**D**) Correlation analysis of miR-106b/miR-93 and PTENP1 expression using Pearson's test. Data are presented as 2^−ΔΔCT^. (**E**–**F**) Correlation analysis of miR-106b/miR-93 and PTEN expression using Pearson's test. Data are presented as 2^−ΔΔCT^.

In order to validate the existence of PTENP1∼ miR-106b/miR-93∼PTEN ceRNA network *in vivo*, we analyzed the correlation of expression of miR-106b/miR-93 and PTENP1/PTEN using clinical GC samples. First, we determined the levels of miR-106b and miR-93 in 36 GC samples relative to the paired normal controls. As we have previously shown, the expression levels showed a significant increase in miR-106b and miR-93 in GC compared to normal tissues, coupled with the downregulation of PTENP1 or PTEN expression in the same cohort (Figure 6A–6B, Figure 1A–1B). We then performed correlation analysis using Pearson's test, which showed a negative correlation between miR-106b/miR-93 and PTENP1/PTEN levels in the GC samples, suggesting a possible linkage of the PTENP1∼miR-106b/miR-93∼PTEN ceRNA network *in vivo* (Figure 6C–6F).

## DISCUSSION

Competing endogenous RNAs are defined as a group of transcripts that can cross-regulate each other by competing for the miRNAs by sharing the same miRNA response elements in their 3′ untranslated regions. CeRNAs are usually composed of mRNAs, lncRNAs, and the newly discovered circRNAs. Recent studies found that ceRNAs regulatory networks participate in a variety of biological processes, including the regulation of gene expression in carcinogenesis and cancer progression [3, 13–16]. As a major member of the lncRNA family, a pseudogene is an ideal ceRNA for its ancestral gene due to the high homology seen between them.

PTENP1 possesses an analogous upstream region of 3′UTR to PTEN, which contains perfectly conserved MREs shared by the PTEN-targeting miRNAs. PTENP1 has been reported to modulate PTEN expression via PTENP1∼miRNA∼PTEN ceRNA network, thereby exerting its tumor suppressive functions in a variety of malignancies [3, 6–9, 16, 17]. However, PTEN-associated ceRNA networks have complicated and tightly regulated functions, since subtle changes in PTEN levels can dictate critical outcomes in tumorigenesis and tumor progression *in vivo* [18, 19]. In addition, the expression of ceRNAs itself is highly cell type and context dependent. Thus, whether the PTENP1∼miRNA∼PTEN ceRNA network exists and how it functions, especially in light of the discovery of the miRNA(s) bridging PTENP1 to PTEN ceRNA network in GC, warrant further investigation. In order to identify and characterize the PTENP1∼miRNA∼PTEN ceRNA network, we determined PTENP1 levels in clinical GC biopsies and revealed that PTENP1 and PTEN were concurrently downregulated in the tumor tissues. Furthermore, *in vitro* characterization validated that PTENP1 acted as a ceRNA, sequestering miR-106b and miR-93 from targeting PTEN for downregulation. Thus, PTENP1 modulates PTEN expression via PTENP1∼miR-106b/miR-93∼PTEN ceRNA network, allowing for PTENP1 to play a tumor-suppressive role in GC cells.

MiR-106b and miR-93 belong to miR-106b-25 cluster, which resides in the 13th intron of the DNA replication gene MCM7 on chromosome 7 in human [20]. Overexpression of miR-106b and miR-93 has been reported in various malignancies, including gastric cancer, hepatocellular carcinoma, esophageal adenocarcinoma, neuroblastoma and prostate cancer [21–25]. We have previously reported that overexpression of miR-106b-25 cluster occurs in GC tissues [26]. Our results further showed that suppression of miR-106b-25 cluster expression inhibited the proliferation, migration, and invasion while promoted apoptosis of GC cells, suggesting a pro-oncogenic role of miR-106b-25 cluster in GC [27]. In this study, we uncovered at least one underlying mechanism through which miR-106b-25 cluster exerts its pro-oncogenic functions in GC cells, highlighting a key regulatory role of miR-106b/miR-93 in PTEN ceRNA network.

However, it is important to note that miR-106b/miR-93 also targets a wide range of tumor suppressor transcripts beyond PTEN, such as BIM, p21, and E2F1 [23, 25, 28, 29]. E2F1, for example, it is a strong stimulator of transcription of Mcm7 gene, thereby promoting the expression level of miR-106b and miR-93. Reciprocally, the E2F1 transcript is targeted by miR-106b and miR-93 for downregulation. This creates a negative feedback loop between E2F1 activity and the transcription of the Mcm7 gene (miR-106b/miR-93). Thus, the cellular phenotypes we observed by modulating the PTENP1∼miR-106b/miR-93∼PTEN ceRNA network may be attributed to the breakage of such a negative feedback loop. Conversely, using a combined computational and experimental approach, Tay *et al*. characterized a panel of protein-coding ceRNAs that regulate PTEN level, including NCOA7, BCL11B, SERINC1, ZNF460, NUDT13, DTWD2, and VAPA transcripts, which adds another layer on the complexity of PTEN ceRNA network [13]. Therefore, other targets for miR-106b and miR-93, which impact GC carcinogenesis and progression via PTEN ceRNA network, as well as a broader PTEN ceRNA network need to be further explored.

## MATERIALS AND METHODS

### Patients and tissues

Surgical specimens were collected from 36 GC patients who underwent surgery at Tianjin Medical University Cancer Institute and Hospital. The study was approved by the Human Research Ethics Committee from Tianjin Medical University. Patients were eligible if they met the inclusion criteria, e.g. those patients who had no precious chemotherapy or radiotherapy and pathologically proven gastric adenocarcinoma. The adjacent noncancerous tissues were obtained in the site at least 5 cm away from the edge of tumor. All tissues, including GC and adjacent noncancerous tissues, were preserved in liquid nitrogen and stored at −80°C until use. Written informed consent was taken from every subject.

### Cell lines and culture

The human gastric epithelial mucosa cell GES-1 was cultured in DMEM (Gibco) medium supplemented with 10% fetal bovine serum (FBS). The gastric adenocarcinoma cell line AGS was cultured in F12K containing 10% FBS, SGC7901, MGC803 and BGC823 were grown in RPMI-1640 (Gibco) medium supplemented with 10% FBS. All the cell lines were maintained in a humidified atmosphere of 5% CO_2_ at 37°C.

### Quantitative real-time PCR

Total RNA was isolated from 36 tumor and paired-adjacent normal tissues or culture cells using Trizol (Invitrogen) according to the manufacture's protocol. Reverse transcription reactions were performed using RT-PCR kit (Takara). The quantitative real-time PCR (qRT-PCR) analysis was performed in an Applied Biosystems 7500 PRISMTM by using the SYBR^®^ Green Premix Ex Taq II (TaKaRa). Primer sequences for qRT-PCR were as follows: PTEN forward, 5′-GTTTACCGGCAGCATCAAAT-3′, and PTEN reverse, 5′-CCCCCACTTTAGTGCACAGT-3′; PTENP1 forward, 5′-TCAGAACATGGCATACACCAA-3′, and PTENP1 reverse, 5′-TGATGACGTCCGATTTTTCA-3′. GAPDH was used as the internal reference. The relative quantification of gene expression was calculated with the 2^−ΔΔct^ method. All reactions were performed in triplicate.

### Plasmid construction

The full length of PTENP1 was amplified by PCR using the product brought from Guangzhou Fitgene Biotech Co Ltd as a template. The PCR products were purified with the NucleoSpin^®^ Gel and PCR Clean-up (MACHEREY-NAGEL) and then cloned into lentiviral vector pCDH. The combination was named pCDH-PTENP1. The sequence corresponding to the wild-type PTENP1 3′UTR was amplified by PCR and then inserted in the luciferase reporter plasmid pIS0. The synthetic plasmid was called pIS0-PTENP1. KOD-Plus-Mutagenesis kit (TOYOBO) was used to construct the mutant plasmids (pCDH-mutPTENP1 or pIS0-mutPTENP1) with point mutations in miR-106b/miR-93 binding site. All the above-mentioned constructs were verified by DNA sequencing.

### Lentivirus packaging and cell infection

For the lentiviral packaging, 4 ug △R, 4 ug VSVG and 8 ug the target plasmid (pCDH-PTENP1, pCDH-mutPTENP1 or pCDH) were transfected into 293T cells with Lipofectamine 2000 (Invitrogen). Six hours later, the media containing DNA-liposome complex were removed and replaced the fresh media. Forty-eight hours after transfection, cell supernatants were collected and filtered through a 0.45 um filter membrane before used to infect the GC cell lines.

### Cell proliferation

Cells were transferred into 96-well plates at 3000 cells per well. 20 μl MTT was added at 0, 12, 24, 48, and 72 hours later. After incubating for 4 hours until purple precipitate was visible, the media were removed carefully and 150 μl DMSO was added to each well. Cell proliferation at different time points was determined by using a microplate reader at 490 nm. Each experiment was run in triplicate.

### Cell apoptosis analysis

Cells were grown in six-well plates for 18∼24 hours in 2ml medium at 37°C and then collected. The cells were washed twice with cold PBS and re-suspended in 100ul Binding Buffering (BD Pharmingen). Then 5 ul Annexin V-FITC and 5 ul PI solution were added and the mixture was incubated at room temperature in the dark for 20 minutes. Finally, the stained cells were analyzed by flow cytometry within 30 minutes. Apoptosis rate = early apoptosis (Q4) +late apoptosis (Q2).

### Cell invasion and migration

A density of 1 × 10^5^ cells in serum-free media was replanted in the upper chambers of 24-well plates with either the Matrigel-coated or uncoated. The lower chamber was placed with RPMI-1640 medium containing 10%FBS. After 24 hours of incubating at 37°C with 5% CO2, cells that passed through the pores were fixed in methanol and stained with 0.1% crystal violet and then counted under microscope.

### Luciferase reporter assay

Cells in 96-well plates were co-transfected with 50 ng of pIS0-PTENP1 or pIS0-mutPTENP1 and 0.5 ug of a renilla luciferase vector (pRL-TK) plus miRNA mimics or negative control (RIBOBIO) with Lipofectamine 2000. Twenty-four hours after transfection, firefly and renilla luciferase activities were successively measured using a luciferase assay system (Promega). The relative luciferase activity was calculated as the ratio of firefly luciferase activity versus renilla luciferase activity.

### Transient transfection and ceRNA concentration gradient assay

Cells were seeded in 6-well dishes the night before to give 70–80% confluence at the day of transfection. The following day they were co-transfected with 10nM miRNA mimics (miR-106b, miR-93) or negative control and different dose of pCDH-PTENP1 or pCDH-mutPTENP1(250 ng, 500 ng, 1 ug, 1.5 ug, 2 ug) using Lipofectamine 2000, according to the manufacturer's instructions. After transfection for 6 hours, the medium was replaced with normal culture medium. 48 hours later, cells were harvested and RNA was extracted using Trizol.

### Statistical analysis

Data are expressed as mean ± SD and analyzed using GraphPad Prism 5.0. Student's *t-test* was performed to compare two different groups. Linear regression analysis was used to measure the correlation between PTEN mRNA level and PTENP1 mRNA level. *P* < 0.05 was considered statistically significant.

## SUPPLEMENTARY MATERIALS FIGURES AND TABLE




