# Supplementary material for: Long non-coding RNA PTENP1 functions as a ceRNA to modulate PTEN level by decoying miR-106b and miR-93 in gastric cancer
Source: Oncotarget. 2017 Feb 13;8(16):26079–89. doi: 10.18632/oncotarget.15317 (PMC5432239; doi:10.18632/oncotarget.15317)
Supplement: Supplementary file 1 [file oncotarget-08-26079-s001.pdf]

Long non-coding RNA PTENP1 functions as a ceRNA to modulate PTEN level by decoying miR-106b and miR-93 in gastric cancer

Supplementary Materials

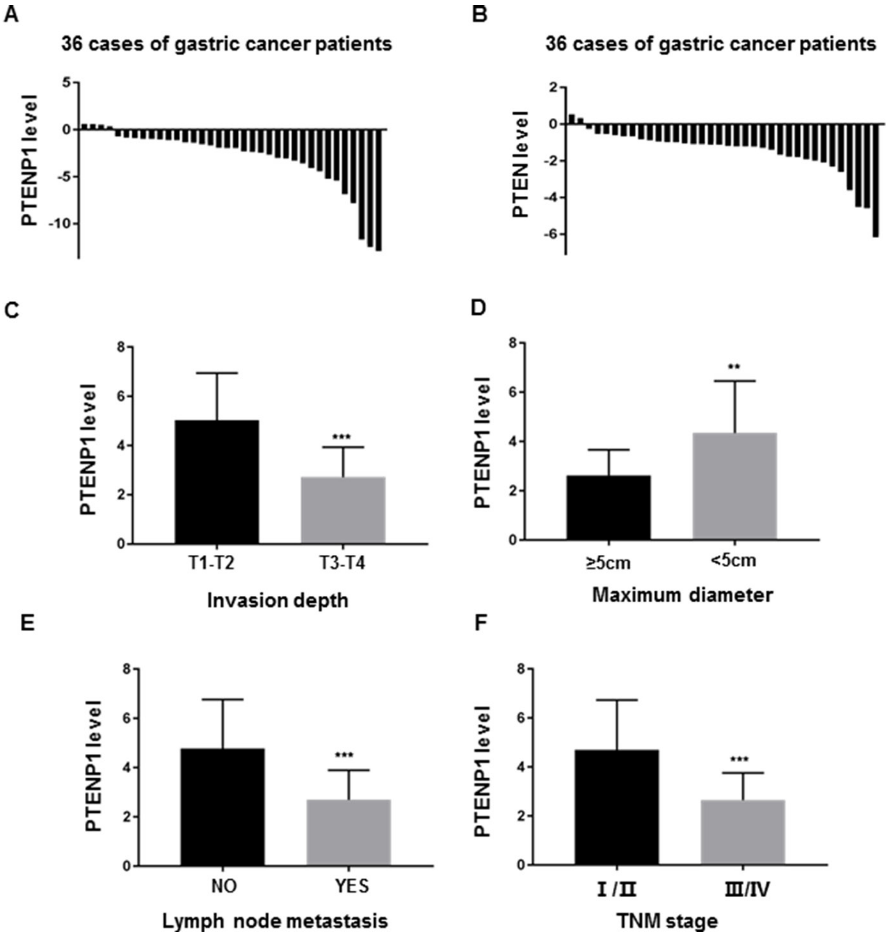

Supplementary Figure 1: Histopathological significance of PTEN and PTENP1 expression in GC. (**A–B**) The expression of PTENP1 and PTEN mRNA in GC tissues relative to paired-normal tissues were determined by qRT-PCR. Data are presented as $2^{-\Delta\Delta CT}$. (**C–F**) PTENP1 mRNA was significantly related to the clinicopathological features, including tumor size, invasion depth, lymph node metastasis and TNM stage. Data are presented as In$2^{-\Delta\Delta CT}$. *P* values were obtained by paired *t*-test (***P* < 0.01; ****P* < 0.001).

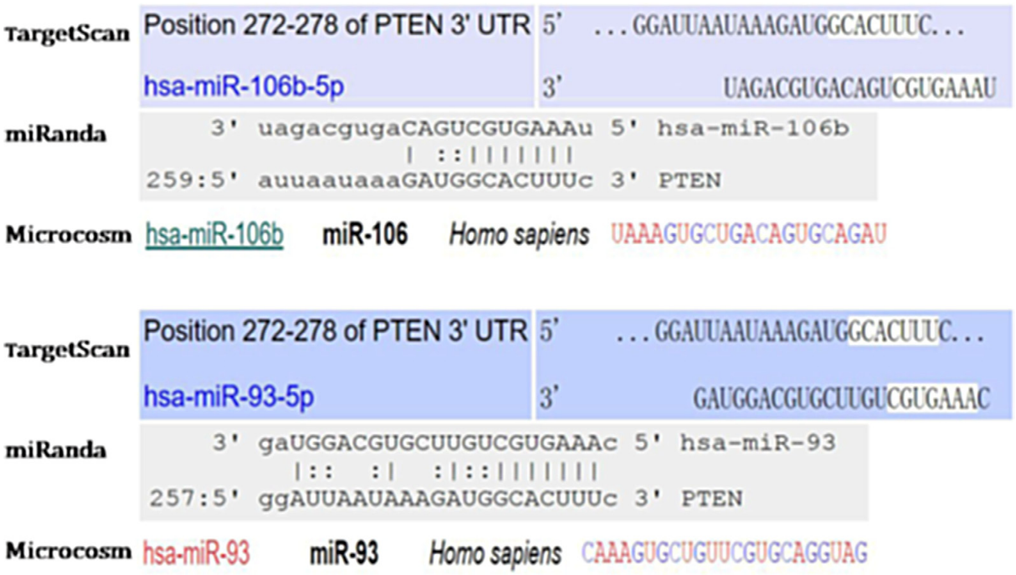

Supplementary Figure 2: Identification of miR-106b/miR-93 that could bind to PTENP1 and PTEN transcripts simultaneously by TargetScan, miRanda and Microcosm Targets bioinformatics software. A seven base-pair sequence at position 272-278 of PTEN 3′UTR was identified to be the seed sequence for miR-106b/miR-93.

Supplementary Table 1: A list of the putative miRNAs for PTENP1-binding. See Supplementary_Table_1
