# Supplementary material for: Long non-coding RNA PTENP1 functions as a ceRNA to modulate PTEN level by decoying miR-106b and miR-93 in gastric cancer
Source: Oncotarget. 2017 Feb 13;8(16):26079–89. doi: 10.18632/oncotarget.15317 (PMC5432239; doi:10.18632/oncotarget.15317)
Supplement: Supplementary file 2 [file oncotarget-08-26079-s002.pdf]

ceRNA	miRNA	mRNA
PTENP1	miR-17/17-5p/20ab/20b-5p/93/106ab/427/518a-3p/519d	SLK
PTENP1	miR-204/204b/211	SLK
PTENP1	miR-219-5p/508/508-3p/4782-3p	SLK
PTENP1	miR-382	SLK
PTENP1	miR-17/17-5p/20ab/20b-5p/93/106ab/427/518a-3p/519d	TRUB1
PTENP1	miR-494	TRUB1
PTENP1	miR-544/544ab/544-3p	TRUB1
PTENP1	miR-590-3p	TRUB1
PTENP1	miR-382	BACH1
PTENP1	miR-494	BACH1
PTENP1	miR-544/544ab/544-3p	BACH1
PTENP1	miR-590-3p	BACH1
PTENP1	miR-17/17-5p/20ab/20b-5p/93/106ab/427/518a-3p/519d	PTEN
PTENP1	miR-382	PTEN
PTENP1	miR-494	PTEN
PTENP1	miR-590-3p	PTEN
PTENP1	miR-219-5p/508/508-3p/4782-3p	UPF2
PTENP1	miR-382	UPF2
PTENP1	miR-494	UPF2
PTENP1	miR-590-3p	UPF2
PTENP1	miR-17/17-5p/20ab/20b-5p/93/106ab/427/518a-3p/519d	TANC1
PTENP1	miR-204/204b/211	TANC1
PTENP1	miR-544/544ab/544-3p	TANC1
PTENP1	miR-590-3p	TANC1
PTENP1	miR-17/17-5p/20ab/20b-5p/93/106ab/427/518a-3p/519d	WDFY3
PTENP1	miR-382	WDFY3
PTENP1	miR-544/544ab/544-3p	WDFY3
PTENP1	miR-590-3p	WDFY3
PTENP1	miR-382	CYB5R4
PTENP1	miR-544/544ab/544-3p	CYB5R4
PTENP1	miR-590-3p	CYB5R4
PTENP1	miR-17/17-5p/20ab/20b-5p/93/106ab/427/518a-3p/519d	FOXJ3
PTENP1	miR-219-5p/508/508-3p/4782-3p	FOXJ3
PTENP1	miR-544/544ab/544-3p	FOXJ3
PTENP1	miR-590-3p	FOXJ3
PTENP1	miR-494	FOXJ3
PTENP1	miR-17/17-5p/20ab/20b-5p/93/106ab/427/518a-3p/519d	VPS13C
PTENP1	miR-204/204b/211	VPS13C
PTENP1	miR-219-5p/508/508-3p/4782-3p	VPS13C
PTENP1	miR-590-3p	VPS13C
PTENP1	miR-17/17-5p/20ab/20b-5p/93/106ab/427/518a-3p/519d	PTGER4
PTENP1	miR-544/544ab/544-3p	PTGER4
PTENP1	miR-590-3p	PTGER4
PTENP1	miR-17/17-5p/20ab/20b-5p/93/106ab/427/518a-3p/519d	IPO5
PTENP1	miR-204/204b/211	IPO5

PTENP1	miR-382	IPO5
PTENP1	miR-494	IPO5
PTENP1	miR-544/544ab/544-3p	IPO5
PTENP1	miR-17/17-5p/20ab/20b-5p/93/106ab/427/518a-3p/519d	BTBD7
PTENP1	miR-590-3p	BTBD7
PTENP1	miR-204/204b/211	BTBD7
PTENP1	miR-544/544ab/544-3p	BTBD7
PTENP1	miR-219-5p/508/508-3p/4782-3p	BTBD7
PTENP1	miR-382	BTBD7
PTENP1	miR-494	BTBD7
PTENP1	miR-204/204b/211	PPIL4
PTENP1	miR-382	PPIL4
PTENP1	miR-494	PPIL4
PTENP1	miR-590-3p	ACSL4
PTENP1	miR-204/204b/211	ACSL4
PTENP1	miR-494	ACSL4
PTENP1	miR-544/544ab/544-3p	ACSL4
PTENP1	miR-17/17-5p/20ab/20b-5p/93/106ab/427/518a-3p/519d	ACSL4
PTENP1	miR-494	ARL5A
PTENP1	miR-204/204b/211	ARL5A
PTENP1	miR-382	ARL5A
PTENP1	miR-17/17-5p/20ab/20b-5p/93/106ab/427/518a-3p/519d	ARL5A
PTENP1	miR-590-3p	ANO6
PTENP1	miR-17/17-5p/20ab/20b-5p/93/106ab/427/518a-3p/519d	ANO6
PTENP1	miR-204/204b/211	ANO6
PTENP1	miR-494	ANO6
PTENP1	miR-544/544ab/544-3p	ANO6
PTENP1	miR-17/17-5p/20ab/20b-5p/93/106ab/427/518a-3p/519d	CCNI
PTENP1	miR-382	CCNI
PTENP1	miR-494	CCNI
PTENP1	miR-590-3p	CCNI
PTENP1	miR-204/204b/211	STX12
PTENP1	miR-382	STX12
PTENP1	miR-494	STX12
PTENP1	miR-590-3p	STX12
PTENP1	miR-17/17-5p/20ab/20b-5p/93/106ab/427/518a-3p/519d	HIF1A
PTENP1	miR-494	HIF1A
PTENP1	miR-590-3p	HIF1A
PTENP1	miR-17/17-5p/20ab/20b-5p/93/106ab/427/518a-3p/519d	SGMS1
PTENP1	miR-494	SGMS1
PTENP1	miR-544/544ab/544-3p	SGMS1
PTENP1	miR-590-3p	SGMS1
PTENP1	miR-17/17-5p/20ab/20b-5p/93/106ab/427/518a-3p/519d	XRN1
PTENP1	miR-204/204b/211	XRN1
PTENP1	miR-494	XRN1
PTENP1	miR-544/544ab/544-3p	XRN1

**PTENP1**	miR-590-3p	XRN1
**PTENP1**	miR-17/17-5p/20ab/20b-5p/93/106ab/427/518a-3p/519d	AFF4
**PTENP1**	miR-544/544ab/544-3p	AFF4
**PTENP1**	miR-204/204b/211	AFF4
**PTENP1**	miR-219-5p/508/508-3p/4782-3p	AFF4
**PTENP1**	miR-382	AFF4
**PTENP1**	miR-494	AFF4
**PTENP1**	miR-590-3p	AFF4
**PTENP1**	miR-17/17-5p/20ab/20b-5p/93/106ab/427/518a-3p/519d	FBXL5
**PTENP1**	miR-204/204b/211	FBXL5
**PTENP1**	miR-382	FBXL5
**PTENP1**	miR-494	FBXL5
**PTENP1**	miR-17/17-5p/20ab/20b-5p/93/106ab/427/518a-3p/519d	ITPRIPL2
**PTENP1**	miR-204/204b/211	ITPRIPL2
**PTENP1**	miR-382	ITPRIPL2
**PTENP1**	miR-544/544ab/544-3p	ITPRIPL2
**PTENP1**	miR-590-3p	ITPRIPL2
**PTENP1**	miR-17/17-5p/20ab/20b-5p/93/106ab/427/518a-3p/519d	LZIC
**PTENP1**	miR-382	LZIC
**PTENP1**	miR-544/544ab/544-3p	LZIC
**PTENP1**	miR-590-3p	LZIC
**PTENP1**	miR-17/17-5p/20ab/20b-5p/93/106ab/427/518a-3p/519d	DEGS1
**PTENP1**	miR-204/204b/211	DEGS1
**PTENP1**	miR-494	DEGS1
**PTENP1**	miR-590-3p	DEGS1
**PTENP1**	miR-17/17-5p/20ab/20b-5p/93/106ab/427/518a-3p/519d	ANKRD17
**PTENP1**	miR-204/204b/211	ANKRD17
**PTENP1**	miR-494	ANKRD17
**PTENP1**	miR-544/544ab/544-3p	ANKRD17
**PTENP1**	miR-590-3p	ANKRD17
**PTENP1**	miR-17/17-5p/20ab/20b-5p/93/106ab/427/518a-3p/519d	RPS6KA5
**PTENP1**	miR-204/204b/211	RPS6KA5
**PTENP1**	miR-494	RPS6KA5
**PTENP1**	miR-544/544ab/544-3p	RPS6KA5
**PTENP1**	miR-17/17-5p/20ab/20b-5p/93/106ab/427/518a-3p/519d	ARAP2
**PTENP1**	miR-204/204b/211	ARAP2
**PTENP1**	miR-590-3p	ARAP2
**PTENP1**	miR-590-3p	SENP6
**PTENP1**	miR-544/544ab/544-3p	SENP6
**PTENP1**	miR-382	SENP6
**PTENP1**	miR-17/17-5p/20ab/20b-5p/93/106ab/427/518a-3p/519d	EYA4
**PTENP1**	miR-590-3p	EYA4
**PTENP1**	miR-544/544ab/544-3p	EYA4
**PTENP1**	miR-382	EYA4
**PTENP1**	miR-204/204b/211	MRPL19
**PTENP1**	miR-544/544ab/544-3p	MRPL19

**PTENP1**	miR-590-3p	MRPL19
**PTENP1**	miR-219-5p/508/508-3p/4782-3p	TMEM135
**PTENP1**	miR-382	TMEM135
**PTENP1**	miR-494	TMEM135
**PTENP1**	miR-590-3p	TMEM135
**PTENP1**	miR-17/17-5p/20ab/20b-5p/93/106ab/427/518a-3p/519d	STX6
**PTENP1**	miR-204/204b/211	STX6
**PTENP1**	miR-382	STX6
**PTENP1**	miR-494	STX6
**PTENP1**	miR-590-3p	STX6
**PTENP1**	miR-17/17-5p/20ab/20b-5p/93/106ab/427/518a-3p/519d	SRPK2
**PTENP1**	miR-204/204b/211	SRPK2
**PTENP1**	miR-494	SRPK2
**PTENP1**	miR-590-3p	SRPK2
**PTENP1**	miR-17/17-5p/20ab/20b-5p/93/106ab/427/518a-3p/519d	YIPF4
**PTENP1**	miR-204/204b/211	YIPF4
**PTENP1**	miR-382	YIPF4
**PTENP1**	miR-544/544ab/544-3p	YIPF4
**PTENP1**	miR-590-3p	YIPF4
**PTENP1**	miR-590-3p	PPM1B
**PTENP1**	miR-494	PPM1B
**PTENP1**	miR-382	PPM1B
**PTENP1**	miR-17/17-5p/20ab/20b-5p/93/106ab/427/518a-3p/519d	UBE3A
**PTENP1**	miR-204/204b/211	UBE3A
**PTENP1**	miR-219-5p/508/508-3p/4782-3p	UBE3A
**PTENP1**	miR-17/17-5p/20ab/20b-5p/93/106ab/427/518a-3p/519d	USP47
**PTENP1**	miR-204/204b/211	USP47
**PTENP1**	miR-494	USP47
**PTENP1**	miR-590-3p	USP47
**PTENP1**	miR-204/204b/211	KIAA0947
**PTENP1**	miR-544/544ab/544-3p	KIAA0947
**PTENP1**	miR-590-3p	KIAA0947
**PTENP1**	miR-17/17-5p/20ab/20b-5p/93/106ab/427/518a-3p/519d	DENND5B
**PTENP1**	miR-494	DENND5B
**PTENP1**	miR-219-5p/508/508-3p/4782-3p	DENND5B
**PTENP1**	miR-544/544ab/544-3p	DENND5B
**PTENP1**	miR-590-3p	DENND5B
**PTENP1**	miR-17/17-5p/20ab/20b-5p/93/106ab/427/518a-3p/519d	EFNB2
**PTENP1**	miR-219-5p/508/508-3p/4782-3p	EFNB2
**PTENP1**	miR-494	EFNB2
**PTENP1**	miR-544/544ab/544-3p	EFNB2
**PTENP1**	miR-590-3p	EFNB2
**PTENP1**	miR-17/17-5p/20ab/20b-5p/93/106ab/427/518a-3p/519d	ERO1LB
**PTENP1**	miR-204/204b/211	ERO1LB
**PTENP1**	miR-494	ERO1LB
**PTENP1**	miR-590-3p	ERO1LB

**PTENP1**	miR-17/17-5p/20ab/20b-5p/93/106ab/427/518a-3p/519d	GRPEL2
**PTENP1**	miR-219-5p/508/508-3p/4782-3p	GRPEL2
**PTENP1**	miR-382	GRPEL2
**PTENP1**	miR-494	GRPEL2
**PTENP1**	miR-590-3p	GRPEL2
**PTENP1**	miR-17/17-5p/20ab/20b-5p/93/106ab/427/518a-3p/519d	ATP6V1D
**PTENP1**	miR-544/544ab/544-3p	ATP6V1D
**PTENP1**	miR-590-3p	ATP6V1D
**PTENP1**	miR-17/17-5p/20ab/20b-5p/93/106ab/427/518a-3p/519d	ATXN7L3B
**PTENP1**	miR-204/204b/211	ATXN7L3B
**PTENP1**	miR-382	ATXN7L3B
**PTENP1**	miR-544/544ab/544-3p	ATXN7L3B
**PTENP1**	miR-590-3p	ATXN7L3B
**PTENP1**	miR-17/17-5p/20ab/20b-5p/93/106ab/427/518a-3p/519d	SLTM
**PTENP1**	miR-494	SLTM
**PTENP1**	miR-544/544ab/544-3p	SLTM
**PTENP1**	miR-204/204b/211	KHDRBS1
**PTENP1**	miR-494	KHDRBS1
**PTENP1**	miR-544/544ab/544-3p	KHDRBS1
**PTENP1**	miR-17/17-5p/20ab/20b-5p/93/106ab/427/518a-3p/519d	MAK16
**PTENP1**	miR-544/544ab/544-3p	MAK16
**PTENP1**	miR-590-3p	MAK16
**PTENP1**	miR-17/17-5p/20ab/20b-5p/93/106ab/427/518a-3p/519d	SUV39H2
**PTENP1**	miR-382	SUV39H2
**PTENP1**	miR-494	SUV39H2
**PTENP1**	miR-590-3p	SUV39H2
**PTENP1**	miR-382	ZNF639
**PTENP1**	miR-590-3p	ZNF639
**PTENP1**	miR-494	ZNF639
**PTENP1**	miR-17/17-5p/20ab/20b-5p/93/106ab/427/518a-3p/519d	ZNF180
**PTENP1**	miR-204/204b/211	ZNF180
**PTENP1**	miR-544/544ab/544-3p	ZNF180
**PTENP1**	miR-219-5p/508/508-3p/4782-3p	ZDHHC2
**PTENP1**	miR-382	ZDHHC2
**PTENP1**	miR-544/544ab/544-3p	ZDHHC2
**PTENP1**	miR-590-3p	ZDHHC2
**PTENP1**	miR-17/17-5p/20ab/20b-5p/93/106ab/427/518a-3p/519d	MYNN
**PTENP1**	miR-494	MYNN
**PTENP1**	miR-590-3p	MYNN
**PTENP1**	miR-17/17-5p/20ab/20b-5p/93/106ab/427/518a-3p/519d	C17orf51
**PTENP1**	miR-590-3p	C17orf51
**PTENP1**	miR-204/204b/211	C17orf51
**PTENP1**	miR-382	C17orf51
**PTENP1**	miR-494	C17orf51
**PTENP1**	miR-544/544ab/544-3p	C17orf51
**PTENP1**	miR-17/17-5p/20ab/20b-5p/93/106ab/427/518a-3p/519d	SOCS6

PTENP1	miR-590-3p	SOCS6
PTENP1	miR-204/204b/211	SOCS6
PTENP1	miR-382	SOCS6
PTENP1	miR-494	SOCS6
PTENP1	miR-544/544ab/544-3p	SOCS6
PTENP1	miR-17/17-5p/20ab/20b-5p/93/106ab/427/518a-3p/519d	PPP1R3B
PTENP1	miR-494	PPP1R3B
PTENP1	miR-544/544ab/544-3p	PPP1R3B
PTENP1	miR-590-3p	PPP1R3B
PTENP1	miR-17/17-5p/20ab/20b-5p/93/106ab/427/518a-3p/519d	RAB12
PTENP1	miR-494	RAB12
PTENP1	miR-590-3p	RAB12
PTENP1	miR-17/17-5p/20ab/20b-5p/93/106ab/427/518a-3p/519d	SLC33A1
PTENP1	miR-382	SLC33A1
PTENP1	miR-494	SLC33A1
PTENP1	miR-590-3p	SLC33A1
PTENP1	miR-494	BRMS1L
PTENP1	miR-17/17-5p/20ab/20b-5p/93/106ab/427/518a-3p/519d	BRMS1L
PTENP1	miR-590-3p	BRMS1L
PTENP1	miR-544/544ab/544-3p	BRMS1L
PTENP1	miR-544/544ab/544-3p	LPAR1
PTENP1	miR-382	LPAR1
PTENP1	miR-204/204b/211	LPAR1
PTENP1	miR-17/17-5p/20ab/20b-5p/93/106ab/427/518a-3p/519d	SCML2
PTENP1	miR-204/204b/211	SCML2
PTENP1	miR-382	SCML2
PTENP1	miR-494	SCML2
PTENP1	miR-17/17-5p/20ab/20b-5p/93/106ab/427/518a-3p/519d	B3GNT5
PTENP1	miR-204/204b/211	B3GNT5
PTENP1	miR-494	B3GNT5
PTENP1	miR-590-3p	B3GNT5
PTENP1	miR-204/204b/211	FAM168B
PTENP1	miR-382	FAM168B
PTENP1	miR-494	FAM168B
PTENP1	miR-544/544ab/544-3p	FAM168B
PTENP1	miR-590-3p	FAM168B
PTENP1	miR-17/17-5p/20ab/20b-5p/93/106ab/427/518a-3p/519d	ZNF695
PTENP1	miR-382	ZNF695
PTENP1	miR-544/544ab/544-3p	ZNF695
PTENP1	miR-590-3p	ZNF695
PTENP1	miR-17/17-5p/20ab/20b-5p/93/106ab/427/518a-3p/519d	HOOK1
PTENP1	miR-494	HOOK1
PTENP1	miR-204/204b/211	HOOK1
PTENP1	miR-590-3p	HOOK1
PTENP1	miR-17/17-5p/20ab/20b-5p/93/106ab/427/518a-3p/519d	DMTF1
PTENP1	miR-204/204b/211	DMTF1

**PTENP1**	miR-590-3p	DMTF1
**PTENP1**	miR-17/17-5p/20ab/20b-5p/93/106ab/427/518a-3p/519d	CHRNA5
**PTENP1**	miR-382	CHRNA5
**PTENP1**	miR-494	CHRNA5
**PTENP1**	miR-590-3p	CHRNA5
**PTENP1**	miR-17/17-5p/20ab/20b-5p/93/106ab/427/518a-3p/519d	ZBTB9
**PTENP1**	miR-382	ZBTB9
**PTENP1**	miR-494	ZBTB9
**PTENP1**	miR-590-3p	ZBTB9
**PTENP1**	miR-204/204b/211	PDHX
**PTENP1**	miR-382	PDHX
**PTENP1**	miR-590-3p	PDHX
**PTENP1**	miR-17/17-5p/20ab/20b-5p/93/106ab/427/518a-3p/519d	FJX1
**PTENP1**	miR-204/204b/211	FJX1
**PTENP1**	miR-590-3p	FJX1
**PTENP1**	miR-17/17-5p/20ab/20b-5p/93/106ab/427/518a-3p/519d	GALNT3
**PTENP1**	miR-544/544ab/544-3p	GALNT3
**PTENP1**	miR-494	GALNT3
**PTENP1**	miR-590-3p	GALNT3
**PTENP1**	miR-17/17-5p/20ab/20b-5p/93/106ab/427/518a-3p/519d	KIAA0895
**PTENP1**	miR-204/204b/211	KIAA0895
**PTENP1**	miR-382	KIAA0895
**PTENP1**	miR-590-3p	KIAA0895
**PTENP1**	miR-17/17-5p/20ab/20b-5p/93/106ab/427/518a-3p/519d	CXCL6
**PTENP1**	miR-544/544ab/544-3p	CXCL6
**PTENP1**	miR-590-3p	CXCL6
**PTENP1**	miR-17/17-5p/20ab/20b-5p/93/106ab/427/518a-3p/519d	ZNF367
**PTENP1**	miR-204/204b/211	ZNF367
**PTENP1**	miR-494	ZNF367
**PTENP1**	miR-544/544ab/544-3p	ZNF367
**PTENP1**	miR-494	TBL1X
**PTENP1**	miR-219-5p/508/508-3p/4782-3p	TBL1X
**PTENP1**	miR-204/204b/211	TBL1X
**PTENP1**	miR-17/17-5p/20ab/20b-5p/93/106ab/427/518a-3p/519d	TBL1X
**PTENP1**	miR-544/544ab/544-3p	TBL1X
**PTENP1**	miR-17/17-5p/20ab/20b-5p/93/106ab/427/518a-3p/519d	GATAD1
**PTENP1**	miR-204/204b/211	GATAD1
**PTENP1**	miR-544/544ab/544-3p	GATAD1
**PTENP1**	miR-590-3p	GATAD1
**PTENP1**	miR-17/17-5p/20ab/20b-5p/93/106ab/427/518a-3p/519d	DNM1L
**PTENP1**	miR-544/544ab/544-3p	DNM1L
**PTENP1**	miR-590-3p	DNM1L
**PTENP1**	miR-590-3p	ZNF367
